# Utilizing Electronic Health Records (EHR) and Tumor Panel Sequencing to Demystify Prognosis of Cancer of Unknown Primary (CUP) patients

**DOI:** 10.21203/rs.3.rs-2450090/v1

**Published:** 2023-01-10

**Authors:** Intae Moon, Jaclyn LoPiccolo, Sylvan C. Baca, Lynette M. Sholl, Kenneth L. Kehl, Michael J. Hassett, David Liu, Deborah Schrag, Alexander Gusev

**Affiliations:** 1 Department of Electrical Engineering and Computer Science, Massachusetts Institute of Technology, Cambridge, MA, USA; 2 Department of Medical Oncology, Dana-Farber Cancer Institute, Boston, MA, USA; 3 Center for Functional Cancer Epigenetics, Dana-Farber Cancer Institute, Boston, Massachusetts; 4 Department of Pathology, Brigham and Women’s Hospital, Harvard Medical School, Boston, MA, USA; 5 Division of Population Sciences, Dana-Farber Cancer Institute and Harvard Medical School, Boston, MA, USA; 6 The Broad Institute of MIT & Harvard, Cambridge, MA, USA; 7 Memorial Sloan Kettering Cancer Center, New York, NY, USA; 8 Division of Genetics, Brigham and Women’s Hospital and Harvard Medical School, Boston, MA, USA

## Abstract

Cancer of unknown primary (CUP) is a type of cancer that cannot be traced back to its original site and accounts for 3-5% of all cancers. It does not have established targeted therapies, leading to poor outcomes. We developed OncoNPC, a machine learning classifier trained on targeted next-generation sequencing data from 34,567 tumors from three institutions. OncoNPC achieved a weighted F1 score of 0.94 for high confidence predictions on known cancer types (65% of held-out samples). When applied to 971 CUP tumors from patients treated at the Dana-Farber Cancer Institute, OncoNPC identified actionable molecular alterations in 23% of the tumors. Furthermore, OncoNPC identified CUP subtypes with significantly higher polygenic germline risk for the predicted cancer type and significantly different survival outcomes, supporting its validity. Importantly, CUP patients who received first palliative intent treatments concordant with their OncoNPC-predicted cancer sites had significantly better outcomes (H.R. 0.348, 95% C.I. 0.210 - 0.570, p-value 2.32 × 10^−5^). OncoNPC thus provides evidence of distinct CUP subtypes and offers the potential for clinical decision support for managing patients with CUP.

## Introduction

When a standardized diagnostic work-up, including radiology and pathology review, fails to locate the primary site of a metastatic cancer, it is diagnosed as a cancer of unknown primary (CUP). CUP represents about 3-5% of all cancers worldwide [1] and is characterized by aggressive progression and poor prognosis (survival of 6 to 16 months [2]). The hidden nature of the primary cancer types for a CUP limits treatment options since clinical responses to some treatments are known to vary based on patients’ tumor types (e.g., identical BRAF V600 mutations targetable in melanoma but no colorectal cancer[3]). Emerging cancer treatments targeting actionable molecular alterations are typically developed for specific cancer types: HER2 in breast cancer and EGFR mutation or ALK/ROS1 rearrangement in Non-small cell lung cancer (NSCLC) [4], and are thus inaccessible to CUP patients. Accurately identifying the latent primary site for CUPs and demonstrating clinical benefit from site-specific therapies may thus open many existing treatment options for patients with CUP.

Pathology review plays a key role in determining primary cancer types of malignant tumors based on immunohistochemistry (IHC) results as well as tumor morphology and clinical findings; however, pathological diagnosis can be challenging for highly metastatic or poorly differentiated tumors. For known cancer types, prior studies showed that an IHC-based diagnostic work-up correctly identified 77 - 86% of primary tumors, which further decreased to 60 - 71% for metastatic tumors [5]. For patients with CUP, IHC results suggestive of a single primary diagnosis account for only 25% of tumors [2]. The subjective nature of pathological interpretation and guidelines, as well as the variability in IHC staining techniques across institutions thus makes it challenging to establish consistent protocols for CUP diagnosis [6].

Molecular tumor profiling has been proposed as an alternative for CUP primary classification due to its quantitative nature and high accuracy on tumors with known cancer types [7–11]. Such tools rely on microarray DNA methylation [7], whole genome sequencing (WGS) [8, 11], or RNA-seq data [10] to train machine learning classifiers using reference data from known-primary tumors. However, molecular sequencing remains prohibitive and not integrated into the existing standard of care, limiting the translational potential of such methods. Recently, key work by Penson et al. [9] demonstrated that accurate primary cancer type classifications could be made from next generation sequencing (NGS) of targeted panels, now routinely collected at many cancer centers and applicable to hundreds of thousands of tumors [12]. However, its clinical utility in diagnosing and aiding treatment for patients with CUP was not systematically investigated.

Several recent studies have investigated the potential clinical benefit of molecular CUP classification, in non-randomized prospective studies [13–15] as well as the randomized clinical trials [16]. These trials have often struggled to recruit sufficient numbers of representative patients and explore the full range of available therapies. A recent randomized phase II trial [16] did not find significant improvement in 1-year survival for the treatment group receiving site-specific therapy guided by molecular profiling. However, this study was limited by a small number of patients (n = 101) recruited over 7 years, with few common solid tumor types and well-established therapies [17]. Assessing the clinical benefits of molecular CUP classification thus poses both an opportunity for precision medicine and a major challenge for conventional randomized studies.

In contrast to prospective trials, retrospective Electronic Health Records (EHR) data can capture a larger and more heterogeneous patient population, despite potential biases due to informative missingness and unobserved heterogeneity. Coupling EHR data with tumor sequencing can offer insights into the molecular mechanisms of CUPs and their relationship to clinical outcomes. As panel sequencing is often part of the standard of care, such insights also have the potential to assist diagnostic efforts and clinical management within existing molecular workflows. Here, we utilized multi-center, Next Generation Sequencing (NGS) targeted panel sequencing data from 36,445 tumor samples with known primary cancers to train and evaluate a machine learning classifier predicting a primary cancer type of a given tumor sample. We applied this classifier, named *OncoNPC* (**Onco**logy **N**GS-based **P**rimary cancer type **C**lassifier), to 971 patients with CUP with clinical follow up at the Dana-Farber Cancer Institute (DFCI). Using the OncoNPC cancer type predictions, we identified CUP subtypes that shared specific characteristics with their corresponding predicted primaries including: significant differences in clinical outcomes, elevated germline risk, and prognostic somatic alterations. 23% of OncoNPC classified CUP tumors had actionable somatic variants enabled by their corresponding OncoNPC cancer type predictions. Finally, using EHR-based treatment and survival data, we showed that site-specific treatments concordant with the OncoNPC cancer type predictions led to longer survival than those discordant with the cancer type predictions. Our findings suggest that many CUPs can be classified into meaningful subtypes with the potential to aid clinical decision making.

## Results

### OncoNPC accurately classifies 22 known cancer types

**Figure 1:**
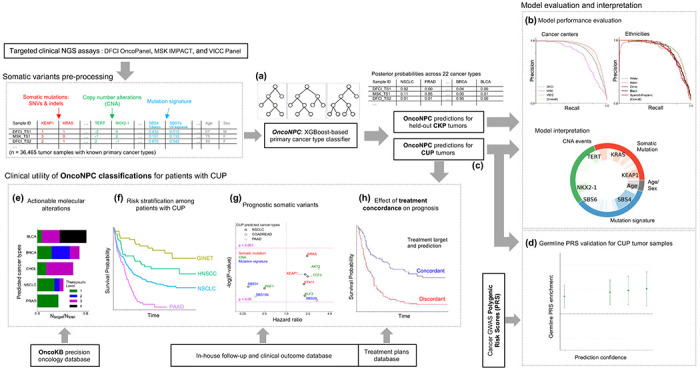
Overview of model development and analysis workflow. **(a)** OncoNPC, a XGBoost-based classifier, was trained and evaluated using 36,729 tumor samples across 22 cancer types from Cancers of Known Primary (CKP) collected from three different cancer centers. **(b)** OncoNPC performance was evaluated on the held-out tumor samples (n = 7,289). **(c)** OncoNPC was applied to 971 CUP tumor samples at a single institution to predict primary cancer types. OncoNPC predicted CUP subtypes were then investigated for association with: **(d)** elevated germline risk, **(e)** actionable molecular alterations, **(f)** overall survival, and **(g)** prognostic somatic features. **(h)** A subset of CUP patients with detailed treatment data were evaluated for treatment-specific outcomes.

**Figure 2:**
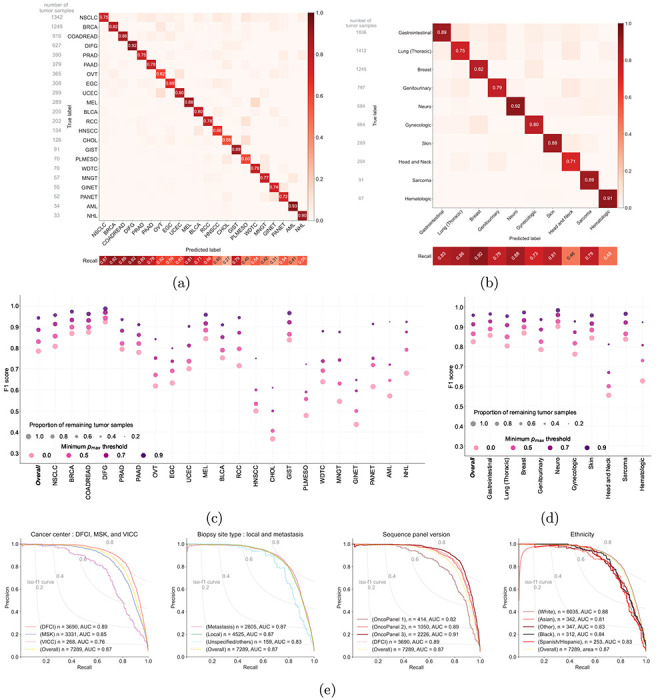
Cancer type prediction performance of OncoNPC. **(a),(b)** The normalized confusion matrix of OncoNPC classification performance on the held-out test set (n = 7,289) for **(a)** 22 detailed cancer types and **(b)** 10 broad cancer groups based on site and treatment (see Table 1). The sensitivity for each cancer type or cancer group is shown below each confusion matrix and the sample size is shown to the left of each confusion matrix. **(c), (d)** The performance of OncoNPC in weighted F1 score on the test set across cancer types **(c)** and groups **(d)** at 4 different prediction confidences (i.e., minimum *p*_*max*_ thresholds). Each dot size is scaled by the proportion of tumor samples retained. **(e)** The precision-recall curves showing OncoNPC’s performance on the test set when grouped by cancer center, biopsy site type, sequence panel version, and ethnicity. The yellow dotted curve represents the baseline performance across the entire test set.

We developed *OncoNPC* (**Onco**logy **N**GS-based **P**rimary cancer type **C**lassifier), a molecular cancer type classifier trained on multicenter targeted panel sequencing data (Fig. 1). OncoNPC utilized all somatic alterations including mutations (single nucleotide variants and indels), mutational signatures, copy number alterations, as well as patient age at sequencing and sex to jointly predict cancer type using a XGBoost algorithm (see Methods). Importantly, no other aspects of tumor morphology, pathology, or patient demographics were used so as not to bias the classifier towards known cancers. OncoNPC was trained and validated on the processed data consisting of 29,176 primary and metastasis tumor samples from 22 known cancer types collected at the DFCI, MSK, and VICC (see Table 1 for details). Across all 22 cancer types, OncoNPC achieved a weighted F1 score of 0.784 on the held-out test tumor samples consisting of 7,289 tumor samples (weighted precision and recall : 0.789 and 0.791, respectively). Across 10 cancer groups (grouped by sites and treatment options (Table 1), OncoNPC achieved an overall weighted F1 score of 0.824 (weighted precision and recall : 0.829 and 0.826, respectively). Despite the evident class imbalance across cancer types, OncoNPC showed well-balanced precision across the cancer types (Fig. 2a) and cancer groups (Fig. 2b). Thresholding on prediction confidence (*p*_*max*_, the maximum posterior probability across all labels) further increased the performance: weighted F1 score of 0.830 with 91.6 % remaining samples at *p*_*max*_ ≥ 0.5 and 0.942 with 65.2 % remaining samples at *p*_*max*_ ≥ 0.9 (Fig. 2c, 2d). While rarer cancer types had generally lower overall performance, increasing the *p*_*max*_ threshold reduced this difference between common/rare cancer types (Fig. 2c, 2d). At *p*_*max*_ ≥ 0, common cancer types in the upper quartile in terms of the number of tumor samples (NSCLC, BRCA, COADREAD, DIFG, PRAD, and PAAD) had a mean F1 of 0.84 while rare cancer types in the lower quartile (WDTC, MNGT, GINET, PANET, AML, and NHL) had a mean F1 of 0.58, whereas at *p*_*max*_ ≥ 0.9 common and rare cancer had a mean F1 of 0.95 and 0.86, respectively. This demonstrates that the OncoNPC was still able to do high-quality predictions for a subset of tumor samples in rare cancer types, for which training data was limited.

OncoNPC achieved robust performance against potential dataset shifts due to the factors including cancer center, biopsy site type, sequence panel version, and patient ethnicity (Fig. 2e). OncoNPC showed comparable performance for tumor samples from DFCI (AUC-PR, area under the precision recall curve = 0.89, n = 3,690) and those from MSK (AUC-PR = 0.85, n = 3,331). OncoNPC performance for those from VICC was slightly lower (AUC-PR = 0.76, n = 268). OncoNPC showed comparable performance for primary tumor samples (AUC-PR = 0.87, n = 4,525) and metastatic tumor samples (AUC-PR = 0.87, n = 2,605), demonstrating its capability to predict the primary cancer site of metastatic cancers without loss of performance. To assess the OncoNPC performance over time, we investigated its performance across sequence panel versions utilized at DFCI, as the panel version is a proxy for sequence dates of tumor samples (see Table 1). The OncoNPC performance on tumor samples from earlier versions of DFCI sequence panels (OncoPanel v1 : AUC-PR = 0.82, n = 414 and OncoPnael v2 : AUC-PR = 0.89, n = 1,050) was slightly lower than the performance on the tumor samples from the most recent panel (OncoPanel v3 : AUC-PR = 0.91, n = 2,226) which also contained the largest number of genes. As all tumor samples have been collected from OncoPanel v3 since October 2016, we expect our model to make high-quality predictions in a prospective setting. Finally, OncoNPC demonstrated consistent performance across patient ethnicity, an important consideration to avoid introducing algorithmic disparities. See Supplementary Fig. S1a for more detailed center-specific OncoNPC performance.

### Applying OncoNPC to CUP tumor samples

We applied OncoNPC to classify 971 CUP tumors from patients who were admitted to DFCI and sequenced as part of routine clinical care. Compared to the held-out cohort of Cancer with Known Primary (CKP; n = 7,289), OncoNPC classifications for CUPs had prediction probabilities lower than those of the DFCI held-out cohort of Cancer with Known Primary (CKP; n = 3,690), but comparable to those of the DFCI held-out cohort of CKPs including other cancer types (n = 8,025), indicating that CUPs may contain other hard-to-classify cancer types: mean prediction probability 0.764 (95% C.I. 0.750 - 778) for CUPs versus 0.881 (95% C.I. 0.875 - 0.887) for the held-out CKPs at DFCI and 0.769 (95% C.I. 0.764 - 0.774) for all held-out CKPs at DFCI (Fig. S1 and Supplementary Fig. S1b). Furthermore, we tested OncoNPC on CKP tumor samples with cancer types that the model was not trained on (i.e., negative control), and the prediction confidence was significantly lower for this group (mean prediction probability 0.674 95% C.I. 0.667 - 0.681; see Supplementary Fig. S1b). This demonstrates that OncoNPC is resistant to making overconfident wrong predictions.

**Figure 3:**
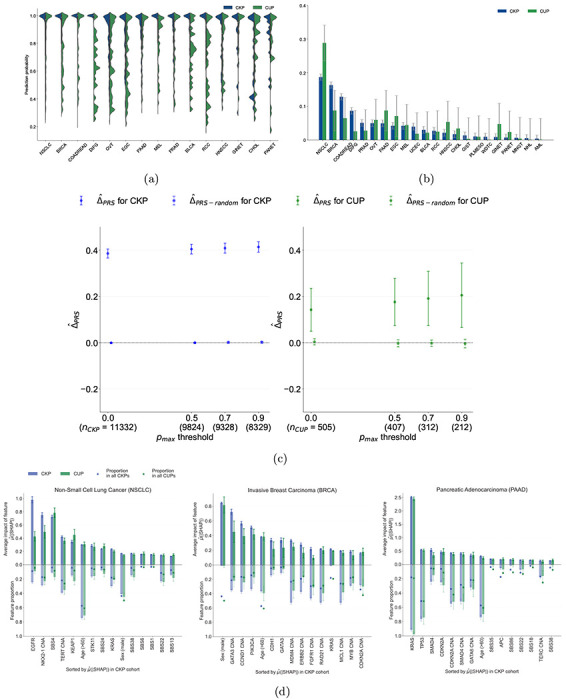
Applying OncoNPC to CUP tumor samples and interpreting cancer type predictions. **(a)** Empirical distributions of prediction probabilities for correctly predicted, held-out CKP tumor samples (n = 3,429) and CUP tumor samples (n = 934) across CKP cancer types (blue) and their corresponding OncoNPC predicted cancer types for CUP tumors (green). Only OncoNPC classifications with at least 20 CUP tumor samples are shown. **(b)** Proportion of each CKP cancer type and the corresponding OncoNPC predicted CUP cancer type. All training CKP tumor samples (n = 36,445) and all held-out CUP tumor samples (n = 971) are included. For both **(a)** and **(b)**, the cancer types (x-axis) are ordered by the number of CKP tumor samples in each cancer type. **(c)** Germline Polygenic Risk Score (PRS) enrichment of the CKP tumor samples (n = 11,332) and CUP tumor samples with available PRS data (n = 505) averaged across 8 cancer types. The magnitude of the enrichment is quantified by ${\hat{\Delta }}_{\text{PRS}}:$ the mean difference between the concordant (i.e., OncoNPC matching) cancer type PRS and mean of PRSs of discordant cancer types (see Methods). ${\hat{\Delta }}_{\text{PRS}}$ is shown for CKPs in blue (for reference) and CUPs in green. As a negative control, ${\hat{\Delta }}_{\text{PRS-random}}$ is also shown after permuting the OncoNPC labels. **(d)** Top 15 most important features based on mean absolute SHAP values (i.e., $\hat{\mu }\left(\left|\text{SHAP}\right|\right)$ [19]) for the top 3 most frequent cancer types in the cohort: Non-Small Cell Lung Cancer (NSCLC), Invasive Breast Carcinoma (BRCA), and Pancreatic Adenocarcinoma (PAAD). The carrier rate for each feature in corresponding CKP and CUP cancer cohorts as well as the entire CKP and CUP cohorts are shown as bars going downwards and star-shaped markers, respectively. For mutation signature features that have continuous values, individuals with feature values one standard deviation above the mean were treated as positives and the rest as negative. For age, individuals above the population mean were treated as positives and the rest as negatives.

Despite the slightly lower prediction confidence, over half of the CUP tumors (518 out of 971) could still be classified with high confidence (i.e., prediction probability > 0.8), and multiple classified types had distributions of posterior probabilities comparable to their corresponding CKPs: Non-small Cell Lung Cancer (NSCLC), Invasive Breast Carcinoma (BRCA), Pancreatic Adenocarcinoma (PAAD), Prostate Adenocarcinoma (PRAD), and Gastrointestinal Neuroendocrine Tumors (GINET). Interestingly, CUPs with predicted GINET were highly confident, despite their small number of tumor samples in the training cohort (n = 359; 0.99% of the training cohort), suggesting some rarer cancer types may nevertheless be confidently identifiable. As shown in Fig. 3b, the most common CUP cancer types were Non-small Cell Lung Cancer (NSCLC), Pancreatic Adenocarcinoma (PAAD), Invasive Breast Carcinoma (BRCA), Esophagogastric Adenocarcinoma (EGC), and Colorectal Adenocarcinoma (COADREAD); of which NSCLC, BRCA, and COADREAD were also the most common CKP types. These rates are broadly consistent with prior findings that the most frequently revealed underlying primary cancers for CUPs by autopsy include lung, large bowel, and pancreas cancers [18]. Finally, comparable rates were observed upon applying OncoNPC to 581 CUP tumors at MSK (Supplementary Fig. S5)

### Explaining OncoNPC cancer type predictions

OncoNPC learns complex non-linear relationships between input somatic variants and clinical features and provides interpretable primary cancer type predictions, where impact of each input feature on a prediction is quantified as a SHAP value [19]. We investigated the most impactful features in predicting each cancer type across the CKP and CUP cohorts to evaluate face validity of OncoNPC (see Fig. 3d for the top 3 most frequent cancer types in the cohort: NSCLC, BRCA, and PAAD, and Supplementary Fig. S2 and S3 for other cancer types). For NSCLC, the most important features were EGFR mutation and SBS4, a tobacco smoking-associated mutation signature [20], for CKP tumor samples and CUP with NSCLC predicted tumor samples, respectively; both consistent with the known etiology of lung cancer. Somatic mutation in the EGFR gene is frequently observed in NSCLC tumors and the gene itself is a well-known therapeutic target for patients with NSCLC [21, 22]. Carcinogens in tobacco smoke have been known to cause lung cancer [23]. For BRCA, the most important feature for both CKP and CUP was sex, as expected, followed by CNA events in GATA3 and CCND1 genes, known drivers and prognostic indicators in breast cancer [24, 25]. For PAAD, KRAS mutation was significantly more common than the population averages and by far the most important somatic feature. Mutations in the KRAS gene occur frequently among patients with colorectal cancer and are known to have prognostic significance [26, 27]. OncoNPC provides intuitive visualizations to explain individual-level predictions. As an example, we show how OncoNPC explained the classification of a tumor sample from a 76 year-old male patient with CUP (see Supplementary Fig. S4). The feature interpretation analysis showed that OncoNPC was able to capture cancer-specific signals in somatic mutations and clinical features, both at the individual and cohort level.

### Germline PRS-based validation on CUP tumor samples

We hypothesized that, if OncoNPC was accurately identifying latent primary cancers, the classified CUP cancer types would exhibit increased germline risk for the corresponding cancers. To that end, we imputed common germline variation for each CUP patient and quantified their polygenic risk scores (PRS) across 8 common cancers using external cancer GWAS data (see Methods). PRSs are a continuous estimate of the underlying germline liability for a given cancer and orthogonal from the somatic data used to train OncoNPC. As hypothesized, patients with CUP had a significantly higher mean germline PRS for the OncoNPC predicted cancers (Fig. 3c and see Supplementary Fig. S7 for cancer type-specific analysis) compared to other cancer types. The magnitude of the difference (i.e., ${\hat{\text{Δ}}}_{\text{PRS}}$) increased for more confident OncoNPC predictions (${\hat{\text{Δ}}}_{\text{PRS}}=0.142$, 95% C.I. 0.0494 – 0.235, two-sided Wald test p-value: 2.66 × 10^−3^ and ${\hat{\text{Δ}}}_{\text{PRS}}=0.204$, 95% C.I. 0.0655 – 0.344, two-sided Wald test p-value: 3.98 × 10^−3^ at *p*_*max*_ threshold = 0.0 and *p*_*max*_ threshold = 0.9, respectively). As a negative control, the same analysis conducted with randomly shuffled OncoNPC labels showed no enrichment. As a positive control, the same analysis conducted on CKPs, with available imputed PRS (n = 11,332), also demonstrated a highly significant germline enrichment, as expected. Notably, the enrichment for CUPs was in between that of CKPs and random tumors, suggesting that while OncoNPC classified CUPs are genetically correlated with CKPs, they still exhibit additional heterogeneity.

### OncoNPC-based risk stratification among patients with CUP

**Figure 4:**
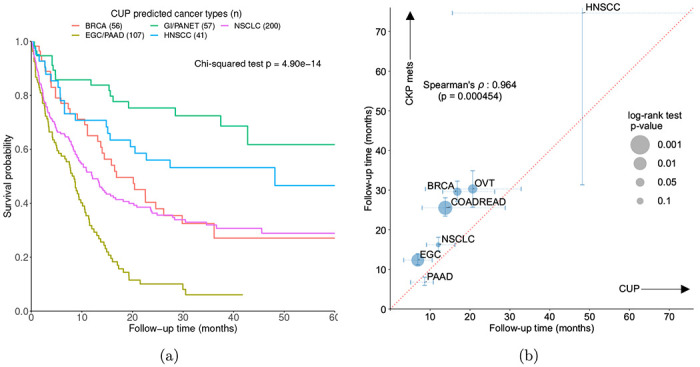
Consistent survival between OncoNPC classifications and known cancers. **(a)** Survival stratification for patients with CUP based on their OncoNPC predicted cancer types. The Kaplan-Meier estimator [55] was used to estimate survival probability for each predicted cancer type over the follow-up time of 60 months from sequence date, with the statistical significance assessed by Chi-square test. **(b)** Correspondence between median survival time (in months) of CUP predicted cancer types (x-axis) and those of metastatic CKP cancer types (y-axis): Spearman’s rho 0.964 (p-value: 4.54 × 10^−4^, Python scipy v1.7.1 [58]). The size of each dot reflects the p-value of the log-rank test for significant difference in median survival between CUP - metastatic CKP pairs. Only cancer types with at least 30 CUP tumor samples having OncoNPC probabilities greater than 0.5 are shown.

To demonstrate clinical utility of OncoNPC, we examined if OncoNPC cancer type predictions can stratify risk among patients with CUP. Using overall survival, we identified subtypes which had significant prognostic differences in median survival based on the OncoNPC classifications (Figure 4a, Chi-squared test, p-value: 4.90 × 10^−14^). Overall, the poorest prognosis was observed in patients with CUP predicted to be Esophagogastric Adenocarcinoma (EGC) and Pancreatic Adenocarcinoma (PAAD): median survival 8.44 months for the combined cohort (95% C.I. 5.39 - 10.5, n = 107). The most favorable prognosis was observed in patients with CUP predicted to be Head and Neck Squamous Cell Carcinoma (HNSCC), Gastrointestinal Neuroendocrine Tumors (GINET), and Pancreatic Neuroendocrine Tumors (PANET): median survival 48.2 months for HNSCC (95% C.I. 19.6 - not estimable, n = 41) and not estimable median survival (i.e. the estimated survival curve never reached the median) for the combined GINET and PANET cohort (n = 57), respectively. Our identified favorable subtypes are consistent with established favorable CUP subtypes such as poorly or well differentiated neuroendocrine carcinomas of unknown primary and squamous cell carcinoma of non-supraclavicular cervical lymph nodes [28]. OncoNPC subtypes can thus be leveraged to meaningfully stratify patients by expected median survival.

### CUP-CKP metastatic survival comparison

We investigated if cancer-specific prognosis is shared between CUP predicted cancer and their corresponding CKP metastatic cancers. Utilizing overall survival data linked to the National Death Index and in-house follow-up data (see Methods), we found that median survival times of CUP-metastatic CKP pairs were significantly correlated across the cancer types (Spearman’s *ρ*: 0.964, p-value: 4.54 × 10^−4^; Fig. 4b). This significant relationship provides evidence that genetics-based OncoNPC predictions capture prognostic signals specific to each predicted cancer type. While correlated, median survival times were significantly lower for patients with CUP compared to those with metastatic CKP: CUP median survival 14.0 months (95% C.I. 11.9 - 15.8, n = 685) vs. metastatic CKP median survival 23.1 months (95% C.I. 21.8 - 24.2, n = 7,797). This is expected as CUPs are an advanced metastatic cancer with limited treatment options [28]. The absolute difference in median survival was significant across all predicted CUP - metastatic CKP pairs with the exception of Pancreatic Adenocarcinoma (CUP PAAD median survival 8.61 months 95% C.I. 5.09 - 10.8 vs. metastatic CKP PAAD median survival 6.73 months 95% C.I. 5.98 - 8.02), known to be a particularly deadly cancer type. Finally, see Supplementary Fig. S8 for prognostic somatic variants shared between OncoNPC CUP subtypes and their corresponding metastatic CKP cancers

### Identifying actionable somatic variants in CUP tumors based on OncoNPC predictions

**Figure 5:**
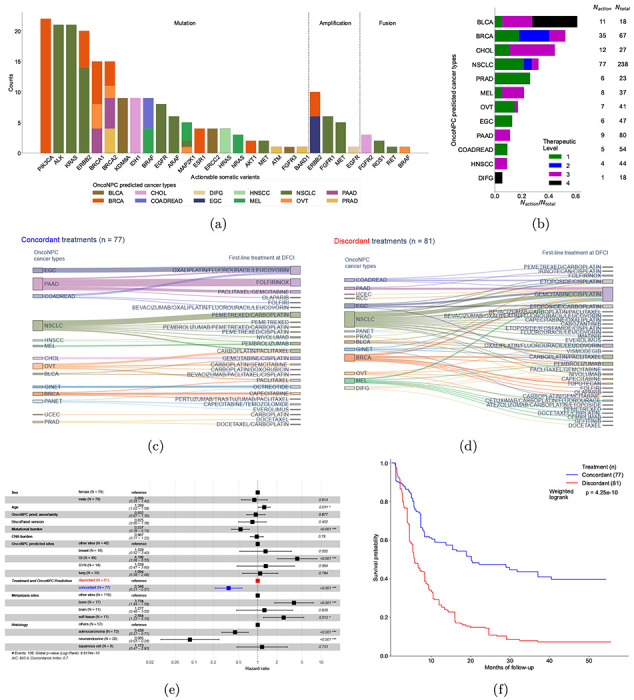
Potential for clinical decision support among OncoNPC classified CUPs. **(a)** The number of CUP tumor samples with actionable targets, based on OncoKB [29], across actionable somatic variants (mutations, amplifications, and fusions). Each bar corresponds to an actionable target, color-coded by the number of each OncoNPC classified CUP carrier. Note that each tumor sample may contain more than one actionable somatic variant. **(b)** Proportions of CUP tumor samples with actionable somatic variants (*N*_*action*_) to the total number of patients (*N_total_*) across OncoNPC predicted cancer types. Proportions for 4 different therapeutic levels based on OncoKB [29], are shown in each bar: Level 1 - FDA-approved drugs, Level 2 – standard of care drugs, Level 3 - drugs supported by clinical evidence, and Level 4 - drugs supported by biological evidence. **(c), (d)** Treatment diagrams for a group of patients with CUP, who received treatments that were concordant with the OncoNPC classification **(c)** and the remaining CUP patients who received discordant treatments **(d)**. OncoNPC classification is shown on the left and treatment groups are shown on the right, with each patient connected from left to right. **(e)** Forest plot of a multivariable Cox Proportional Hazards Regression on patients in the CUP cohort with first-line palliative treatment records at DFCI (n = 158; see Supplementary Fig. S6 for the exclusion criteria). Treatment concordance (colored in blue), encoded as 1 when the first treatment a patient receives at DFCI aligns *concordant* with their corresponding OncoNPC prediction and 0 otherwise, was significantly associated with mortality of patients in the cohort (H.R. 0.348, 95% C.I. 0.210 - 0.570, p-value 2.32 × 10^−5^). **(f)** Estimated survival curves for patients with CUP in the concordant treatment group (shown in blue) and discordant treatment group (shown in red), respectively. To estimate the survival function for each group, we utilized Inverse Probability of Treatment Weighted (IPTW) Kaplan-Meier estimator while adjusting for left truncation until time of sequencing (see Methods). Statistical significance of the survival difference between the two groups was estimated by a weighted log-rank test [59].

We investigated if OncoNPC classifications could identify genetically driven, site-specific treatment options that are typically available for cancers with known primaries. We utilized OncoKB [29] as a knowledge base and considered three different categories of actionable somatic variants: oncogenic mutation, amplification, and fusion (see Methods). OncoNPC cancer type predictions enabled identification of actionable somatic variants across CUP tumor samples (total 22.8% of the eligible CUP tumor samples; see Fig. 5a and Fig. 5b). The majority of actionable somatic variants for patients with CUP were oncogenic mutations (183 counts; 87.1%), followed by amplifications (22 counts; 9.52%) and fusions (7 counts; 3.33%) as shown in Fig. 5a. The four most frequent oncogenic mutations were in PIK3CA, KRAS, ALK, and ERBB2 genes, occurring in CUP tumor samples classified as BRCA (PIK3CA and ERBB2 genes) and NSCLC (KRAS, ALK, and ERBB2 genes). Overall, among the eligible CUPs whose prediction confidences are greater than 0.5 (N = 794; see Supplementary Fig. S6 for more details on the exclusion criteria), OncoNPC predictions identified actionable somatic variants for 11.5% of the CUP tumor samples for Level 1 therapeutic level (FDA-approved drugs), 3.63% for Level 2 (Standard care), 6.64% for Level 3 (Clinical evidence), and 1.00% for Level 4 (Biological evidence), summing up to the total 22.8% of the eligible CUP tumor samples (Fig. 5b).

### Survival benefit of treatment concordance with OncoNPC predictions

We performed retrospective survival analysis to investigate whether patients with CUP achieved clinical benefit when treated in concordance with their OncoNPC classifications. We restricted to a cohort of 158 patients with CUP, received first treatment at DFCI with a palliative intent (see the exclusion criteria in Supplementary Fig. S6). Each case was then manually chart reviewed by a certified oncologist to determine whether the treatment administered was concordant with the OncoNPC prediction per National Comprehensive Cancer Network (NCCN) guidelines or standard of care (see Methods, Fig. 5c, and Fig. 5d). Strikingly, patients with CUP who received first palliative treatments concordant with their OncoNPC predicted cancer types exhibited significantly better survival than those who received discordant treatments as shown in Fig. 5e and 5f (*multivariable Cox regression*: H.R. 0.348, 95% C.I. 0.210 - 0.570, p-value 2.32 × 10^−5^, Proportional Hazard assumption test [30]: Chi-squared test with 17 degrees of freedom p-value 0.156, *IPTW Kaplan-Meier estimator*: weighted log-rank test p-value 4.25× 10^−10^). Finally, after stratifying by OncoNPC predicted cancers and repeating the IPTW Kaplan-Meier analysis, we found that the treatment concordant group had improved survival across cancer cohorts (breast, GI, and others), with the exception of the lung cancer cohort (Supplementary Fig. S9).

We note that as this was not a randomized analysis, a potential concern may be systematic differences between the concordant and discordant groups leading to a significant prognostic but not predictive difference [31]. For example, treatment discordant patients may have systematically more advanced/de-differentiated tumors and thus exhibit poorer survival regardless of their treatment regimen. (see Table 2 for comparison of the two groups across the measured covariates). To minimize biases from potential confounders and move towards a predictive estimate of treatment concordance on patient survival, we adopted two estimation strategies: multivariable Cox regression [32] (i.e., covariate adjustment) and Inverse Probability of Treatment Weighted (IPTW) Kaplan-Meier estimator [33] (see Methods), which have recently been employed to emulate estimates from randomized trials [34, 35]. In both multivariable Cox regression and IPTW Kaplan-Meier estimator strategies, patients treated like their OncoNPC predicted cancer types (i.e. those in the concordant treatment group) consistently showed significantly better survival compared to those in the discordant treatment group. The multivariable Cox regression (Fig. 5e) additionally identified significant hazardous effects of age, gastrointestinal (GI) cancer types predicted by OncoNPC, and bone metastasis (H.R. 1.27, 95% C.I. 1.02 – 1.58, p-value 3.10×10^−2^, H.R. 4.20, 95% C.I. 2.06 – 8.55, p-value 7.78× 10^−5^, and H.R. 3.73, 95% C.I. 1.84 – 7.59, p-value 2.71×10^−4^, respectively), and significantly protective effects of tumor mutational burden (TMB), as well as adenocarcinoma and neuroendocrine tumor group determined by the histopathology results (H.R. 0.537, 95% C.I. 0.388 - 0.742, p-value 1.64 × 10^−4^, H.R. 0.439, 95% C.I. 0.272 - 0.710, p-value 7.85 × 10^−4^ and H.R. 0.0854, 95% C.I. 0.0298 - 0.245, p-value 4.79 × 10^−6^, respectively). In the IPTW Kaplan-Meier analysis, we found that treatment concordance with the OncoNPC prediction was associated with Gastrointestinal (GI) cancer types (coefficient 1.916, 95% C.I. 0.627 - 3.205, p-value 3.57 × 10^−3^), whereas male sex and OncoNPC prediction uncertainty (i.e., entropy of predicted probability distribution over the considered cancer types) were inversely associated with receiving concordant treatment (coefficient −1.259, 95% C.I. −2.283 - −0.234, p-value 1.61×10^−2^, and coefficient −1.693, 95% C.I. −2.458 - −0.927, p-value 1.46×10^−5^) (see Supplementary Fig. S10). These associations with treatment concordance are consistent with likely GI CUPs being more clinically identifiable and low OncoNPC confidence CUPs being less clinically identifiable. We note, however, that the IPTW approach specifically adjusts for these systematic differences when estimating the effect of treatment concordance on survival.

## Discussion

Our work provides unique insights into the genetic and prognostic landscapes of CUP tumor samples by utilizing routinely collected EHR and multicenter NGS tumor panel sequencing data. We have developed OncoNPC, a machine learning model for molecular classification of tumor samples based on the NGS panel data. When evaluated with the held-out multicenter test data, OncoNPC provided robust and interpretable predictions. Applying OncoNPC to CUP tumor samples, we demonstrated that the OncoNPC CUP subtypes showed significantly higher germline PRS risk for their predicted cancer. To our knowledge, this is the first evidence of germline genetic correlation between CUPs and corresponding known primaries, and lends orthogonal support to the molecular classification of CUPs into subtypes. We demonstrated clinical utility of the OncoNPC CUP subtypes by showing significant survival differences across subtypes, and, within subtypes, potentially actionable somatic alterations in 11.5% (Level 1 therapeutic level) and 22.8% (all levels) of tumors. Finally, in a retrospective analysis, we showed that patients with CUP, that had been treated in a consistent manner with their OncoNPC classification, achieved significantly longer survival than those treated in an inconsistent manner (multivariable Cox regression: H.R. 0.348, 95% C.I. 0.210 - 0.570, p-value 2.32×10^−5^). Our findings suggest that CUP tumors share a genetic and prognostic architecture with known cancer types, and may benefit from molecular classification with OncoNPC for prognosis as well as treatment decision-making.

The question of whether CUP tumors consist of heterogeneous latent primaries or are a unique cancer type in and of themselves has been actively investigated [18, 36, 37]. Prior studies have demonstrated accurate classification of known tumors using Whole-Genome Sequencing [11], NGS panels [9], RNA-seq [10], methylation [7], and other platforms [38, 39]. However, these algorithms typically applied classification to metastatic tumors of known types and did not investigate the clinical implications for CUPs at large scale. Moran et al., [7] observed a nominally significant difference in survival between patients with CUP who received site-specific treatments concordant with their molecular primary site predictions and those who received empiric treatments. While promising, it remains unknown whether this difference is due to accurate classification for the site-specific group or systematically worse outcomes for the empirically treated group, which is typically a more challenging patient population [40]. To explicitly distinguish these scenarios, our analysis instead restricted to a CUP cohort wherein all patients received site-specific treatments as the first palliative-intent therapy and estimated a significant survival benefit of concordant treatment vs. discordant treatment (excluding the empirically treated group). Our findings were obtained after adjusting for left-truncation for sequencing time and measured potential confounders through covariate adjustment as well as propensity score weighting, which have been recently employed to mimic clinical trials in Real World data [34, 35]. Although we cannot rule out potential biases from unmeasured confounders, our cohort includes more heterogeneous populations compared to recruited cohorts in randomized controlled trials (RCT), and the proposed intervention (concordant treatment vs. discordant treatment) is challenging to ethically evaluate through RCTs, necessitating the use of retrospective causal inference.

Our study has several limitations. Firstly, although we utilized multicenter NGS tumor panel sequencing data to train OncoNPC model for cancer type prediction, we utilized retrospective EHR data from a single institution for the downstream clinical analyses. As a result, these analyses may be susceptible to systematic ascertainment patterns or biases specific to a tertiary academic cancer center. Replication of our clinical findings in other institutions is thus necessary to generalize our results. Secondly, we considered only the 22 most common cancer types in the cohort as classification labels (68.1 % of all tumor samples at DFCI, and 69.9 % across all three centers). As a result, if a CUP tumor sample harbors a distinct yet not modeled primary cancer type, then the tumor sample will likely have high uncertainty in the prediction (see Supplementary Fig. S1b). Nevertheless, prior work has shown that the majority of resolvable primary sites of CUP tumor samples were from common cancers (e.g., lung, pancreas, and GI) [18], consistent with our findings. As more diverse tumor samples are collected across multiple institutions, our model can be augmented to robustly predict rare cancer types as well. Thirdly, our classifier and analyses relied on data from panel sequencing assays targeting 300-500 genes, which are inherently only sensitive to coding mutations and deep copy number alterations in the targeted genes. Other features captured by whole-genome sequencing or molecular assays may thus achieve better classification performance. Our focus in this work was on assays that are in routine clinical use as those are linked to Real World clinical data and offer the most immediate translational potential.

Our findings strongly suggest that routinely collected targeted tumor panel sequencing data have clinical utility in assisting diagnostic work-up and prognosis, and may additionally inform treatment decisions. To date, clinical sequencing is primarily used for identification of known biomarkers and corresponding clinical trial enrollment [41–44], and our findings additionally support use of panel sequencing for diagnosis. Conventional IHC-based pathology reviews are often unable to identify a primary diagnosis for advanced metastatic tumor samples [2, 5], particularly in community clinics where resources are limited. And in many cases, patients do not receive the complete diagnostic work-up that is recommended for CUPs [45]. As a result, oncologists resort to empiric treatment regimens to treat many patients with CUP [18] even when targeted therapies would otherwise be the standard of care for a corresponding known primary. In future work, we envision a multimodal framework that incorporates molecular sequencing together with patient pathology images [39], physiological data, and clinical notes to directly predict optimal treatment regiments rather than just cancer types. Our work thus paves a way for incorporating routine panel sequencing data into clinical decision support tools for clinically challenging cases.

## Methods

### Patients and tumor samples

We used the next generation sequencing (NGS) targeted panel sequencing data collected at three institutions in routine clinical care as part of the AACR project GENIE [12]: Dana-Farber Cancer Institute (DFCI, n=18,816), Memorial Sloan Kettering Cancer (MSK, n=16,294) center, and Vanderbilt-Ingram Cancer Center (VICC, n=1,335). The collected tumor samples represented 22 different cancer types and included 971 total samples from cancer of unknown primary (CUP). National Death Index (NDI) and clinical death and last clinical appointment records were available for 20,281 DFCI patients (n = 16,376 for CKP and n = 838 for CUP). Demographic details of the patients and tumor samples can be found in Table 1.

The cancer centers, DFCI, MSK, and VICC, were chosen because of similar genomic data characterization of their sequence panels in terms of coverage and alteration types [12]. DFCI samples were sequenced using a custom, hybridization-based panel called OncoPanel which targeted exons of 275-447 genes across three panel versions [12, 43]. MSK samples were sequenced using a custom panel called MSK-IMPACT which targeted 341-468 genes across 3 panel versions [12, 42]. VICC samples were sequenced using custom panels called VICC-01-T5A and VICC-01-T7, which targeted 322 and 429 genes, respectively [12]. All panels were capable of detecting single nucleotide variants (SNVs), small indels, copy number alterations, and structural variants [12].

The DFCI CUP cohort consisted of 971 sequenced tumor samples (from 962 patients) with a cancer diagnosis of CUP and the following detailed cancer type: Adenocarcinoma, Not Otherwise Specified (NOS) (n = 345), Cancer of Unknown Primary, NOS (n = 194), Squamous Cell Carcinoma, NOS (n = 114), Poorly Differentiated Carcinoma, NOS (n = 118), Neuroendocrine Tumor/Carcinoma, NOS (n = 170), Small Cell Carcinoma of Unknown Primary (n = 16), Undifferentiated Malignant Neoplasm (n = 12), and Mixed Cancer Types (n = 2). For downstream clinical analyses, we applied additional exclusion criteria, described in Supplementary Fig. S6.

### Developing OncoNPC cancer type classifier

We used a gradient tree boosting framework (XGBoost [46]) to develop OncoNPC for predicting cancer types from molecular features. In this framework, decision trees for the input features are sequentially added to an existing ensemble of the trees, such that the algorithm fits the new tree to the residuals from the ensembles with regularization on the tree structure. As the trees (a.k.a. weak learners) are added, the model learns optimal weights to combine their predictions and produces the improved outcome from the combined ensemble [46]. Owing to its high performance and scalability, the XGBoost method has been used across a wide range of applications in the healthcare space [47–49].

OncoNPC was trained and evaluated using tumors from 22 known cancer types split into 29,176 training samples and 7,289 test samples. Hyper-parameter selection was conducted using random search [50] with 10-fold cross validation within the training set while utilizing weighted F1 score as an evaluation metric. The optimal hyper-parameters were then selected and the model was evaluated on the held-out test set (n = 7,289). To predict primary sites of CUP tumors, the model was then re-trained on all CKP tumor samples and applied to the CUP tumors to estimate posterior probabilities across the 22 different cancer labels. For each tumor sample, a cancer type with the highest probability was chosen as the predicted primary site.

### Feature selection and OncoNPC model interpretation

The OncoNPC model was trained on somatic variant features from tumor sequencing data, as well as patient age at sequencing and sex. Other demographic/clinical features were intentionally not used so as not to bias the model toward cancer types with more available information. Somatic variant features included: mutations (i.e., single nucleotide variants (SNV) and indels), Copy Number Alteration (CNA) events, and mutational signatures [51]. For each gene, the total count of a somatic mutation (i.e., single nucleotide variants and indels) was encoded as a positive integer feature. The presence of a CNA event for each gene was encoded as a categorical variable with 5 levels: −2 (deep loss), −1 (single-copy loss), 0 (no event), 1 (low-level gain), and 2 (high-level amplification); note that CNA events data for tumor samples from MSK and VICC were encoded as −2 (deep loss), 0 (no event), and 2 (high-level amplification). Each of 60 different mutation signatures was inferred as the dot product of the weights derived from [51] and 96 single base substitutions in a trinucleotide context. The single base substitutions were computed using the deconstructSigs v1.8.0 R library [52]. See Supplementary Material for the full set of features.

To identify important features in the OncoNPC’s predictions, we used the recently proposed feature interpretation tool for tree-based models, called TreeExplainer [19] (Python shap v0.41.0). TreeExplainer uses an efficient polynomial time algorithm (*O*(*TLD*^2^), *T* : number of trees, *L* : number of leaves, *D* : maximum depth) to approximate Shapley values which capture the impact of each feature on each individual model prediction. The Shapley value assigned to each feature is modeled as the average change in the model’s conditional expectation function over all possible feature orderings when introducing the corresponding feature into the model; it is formulated as 𝔼_*S*_[*f*(*X*)|do(*X*_*S*_ = *x_S_*)], where *S* is the set of features, *X* is a random variable for the feature to perturb, and do notation [53] reflects the causal feature perturbation formulation. See [19] for more details on the algorithm and its properties.

Applying TreeExplainer on the model outcome at each fold across the 10-fold cross-fitting procedure, we obtained out-of-sample local explanations for all individual model predictions of primary cancer types. By combining local explanations of correct predictions for each cancer type, we characterized the cancer type in terms of the most important or predictive features based on their Shapley values, which provided insights into the somatic variants and clinical features most relevant to the classification of each cancer type.

### Germline PRS-based validation on CUP tumor samples

To validate the OncoNPC predictions for CUP tumor samples (which do not otherwise have a ground truth), we utilized germline Polygenic Risk Scores (PRS) which were never available to OncoNPC for training. Germline imputation from the off-target tumor sequencing data was conducted as previously described in [54]. Using weights from external GWAS data, we imputed PRS for Non-Small Cell Lung Cancer (NSCLC), Invasive Breast Carcinoma (BRCA), Colorectal Adenocarcinoma (COADREAD), Diffuse Glioma (DIFG), Melanoma (MEL), Ovarian Epithelial Tumor (OVT), Renal Cell Carcinoma (RCC), and Prostate Adenocarcinoma (PRAD). Pearson correlation between the PRS from off-target tumor data versus matched germline SNP array was previously shown to be higher than 0.9 without observable outliers [54].

We hypothesized that germline PRS specific to the underlying primary cancer type of a CUP tumor sample would be enriched in a manner similar to how the PRS specific to CKP tumor sample with the same primary cancer type is enriched. To that end, given the set of 8 different cancer types 𝒞 we have the imputed PRS available for, we first restricted the cohort of CUP tumor samples to those with OncoNPC predictions in $𝒞(N_{\mathrm{CUP},𝒞}=505)$. Then, we obtained standardized germline PRS values for the chosen CUP tumor samples over all the cancer types in 𝒞. Finally, we defined ${\hat{\text{Δ}}}_{\text{PRS}}$ as the estimated mean difference between the PRS specific to the predicted primary cancer type 𝒞 (i.e. concordant PRS; PRS_*C*_) and average of PRSs corresponding to the rest of the cancer types (i.e. discordant PRS; PRS_*D*_, where D∈𝒞∖C) as follows

(1) $${\hat{\text{Δ}}}_{\text{PRS}}=\hat{𝔼}\left[{\text{PRS}}_{C}-{\hat{𝔼}}_{D}\left[{\text{PRS}}_{D}|C\right]\right]=\frac{1}{N_{\text{CUP},𝒞}}\overset{N_{\text{CUP},𝒞}}{\underset{i}{\sum }}\left({\text{PRS}}_{c_{i}}-\frac{1}{\left|𝒞\setminus c_{i}\right|}\underset{d_{i}\in 𝒞\setminus C_{i}}{\sum }{\text{PRS}}_{d_{i}}\right)$$.

As a true positive reference, we repeated the above procedure for the CKP tumor samples. Finally, as a true negative null, we estimated ${\hat{\text{Δ}}}_{\text{PRS–random}}$, where the concordant cancer type was randomly assigned. We then repeated the random assignment 100 times to obtain estimated mean and standard errors.

### Survival function estimation

National Death Index (NDI) and in-house clinical records were available for 20,281 DFCI patients (n = 16,376 for CKP and n = 838 for CUP). A patient’s lost to follow-up date was determined at either the last NDI update date (12/31/2020) or their corresponding last contact date from the in-house records, whichever date is later. A patient’s death date was determined from the in-house records, or the NDI data if the patient was lost to follow-up.

### CUP-metastatic CKP survival comparison

We estimated median survival times of patients across CUP - metastatic CKP pairs using the Kaplan-Meier estimator [55] to account for patients lost to follow-up. For the CUP cohort, we excluded patients with CUP that were lost to follow up at the time of tumor sequencing and those whose primary cancer types were predicted with low probability (see Supplementary Fig. S6). The resulting CUP cohort (n = 685), was then restricted to OncoNPCcancer types with more than 35 CUP patients. For the CKP metastatic cohort, we excluded patients lost to follow up at the tumor sequencing time in the same manner and chose patients with one of the known cancers, where either the biopsy was metastatic or the patient had an ICD-10 code indicative of secondary malignant neoplasms within a year prior to sequencing dates. A total of 521 and 5,937 patients were thus retained from the CUP cohort and metastatic CKP cohort, respectively: Non-Small Cell Lung Cancer (NSCLC; n_CUP_ = 200, n_met-CKP_ = 1,559), Pancreatic Adenocarcinoma (PAAD; n_CUP_ = 80, n_met-CKP_ = 357), Invasive Breast Carcinoma (BRCA; n_CUP_ = 67, n_met-CKP_ = 1,656), Colorectal Adenocarcinoma (COADREAD; n_CUP_ = 54, n_met-CKP_ = 1,198), Head and Neck Squamous Cell Carcinoma (HNSCC; n_CUP_ = 44, n_met-CKP_ = 216), Esophagogastric Adenocarcinoma (EGC; n_CUP_ = 40, n_met-CKP_ = 336), and Ovarian Epithelial Tumor (OVT; n_CUP_ = 36, n_met-CKP_ = 615). Note that patients with CUP, whose predicted cancer type is Gastrointestinal Neuroendocrine Tumors (GINET; n_CUP_ = 39, n_CKP_ = 118), were excluded due to the fact that the estimated survival function for the CUP cohort never reached 50 percent.

### OncoNPC-based risk stratification among patients with CUP

To identify OncoNPC CUP subtypes with significant prognostic differences, we estimated survival functions for 7 common OncoNPC subtypes with more than 35 CUP patients: NSCLC, PAAD, BRCA, HNSCC, EGC, GINET, and Pancreatic Neuroendocrine Tumor (PANET). Patients that were lost to follow up at time of sequencing were again excluded, as were CUPs with an OncoNPC prediction probability lower than 0.5 (i.e., same criteria as the CUP - metastatic CKP survival comparison analysis). We merged subtypes with similar morphology and estimated survival functions: PAAD and EGC; GINET and PANET. To statistically test survival differences between these 5 groups, we utilized Chi-squared test with 4 degrees of freedom.

### Actionable somatic variants in CUP tumors

We estimated the frequency of known, actionable somatic alterations in each OncoNPC CUP subtype using the OncoKB knowledge base [29]. OncoNPC CUP predictions with a probability greater than 0.5 were retained (see Supplementary Fig. S6). We considered 3 different types for somatic variants: oncogenic mutations such as indels, missense mutations, and splice site mutations, amplifications such as high-level amplifications, and finally fusions such as gene-gene and gene-intergenic fusions as specified in OncoKB. For each actionable somatic variant, we assigned one of the four therapeutic levels: level 1 for FDA-approved drugs, level 2 for standard care drugs, level 3 for drugs supported by clinical evidence, and level 4 for drugs supported by biological evidence.

### Estimating impacts of treatment concordance on survival of patients with CUP

We estimated the impact of the concordance between treatment and OncoNPC CUP predictions on a mortality outcome in a retrospective survival analysis. We utilized the in-house patient follow-up and treatment data to identify patients with CUP who received first treatment at DFCI with a palliative intent (Supplementary Fig. S6 for the exclusion criteria). Each patient was reviewed by a trained oncologist to determine whether the OncoNPC predicted cancer type was concordant or discordant with the first line of treatment received, per National Comprehensive Cancer Network (NCCN) guidelines or standard of care, in most reasonable situations, and within the clinical context delineated in the medical record. See Supplementary Methods: *Determining treatment-OncoNPC concordance* for more details, and Supplementary Material for clinical information, including primary cancer diagnosis, biopsy site, and first chemotherapy plan at DFCI, of patients with CUP in the analysis.

As we were interested in the counterfactual causal impact of the OncoNPC-treatment concordance, we utilized the principles of causal inference to account for potential patient heterogeneity and confounding. Specifically, we estimated the effect of treatment concordance specified by the indicator variable, *A*, which was 1 when the first palliative treatment for a patient with CUP was concordant with the corresponding OncoNPC prediction and 0 otherwise. Our analyses make the following identifiability assumptions:

- Conditional ignorability : $A_{i}⫫T_{i}^{a_{i}}|X_{i}$, where *A_i_* ∈ 0, 1. It means that given patient *i*’s a set of covariates *X*_*i*_, the patient’s treatment concordance *A*_*i*_ is as good as random.
- Consistency : $T_{i}^{a_{i}}=T_{i}$, which means that a counterfactual outcome $T_{i}^{a_{i}}$ for patient *i* is the observed outcome for the patient with a treatment concordance *a*_*i*_.
- Overlap : *P*(0 < *p*(*X*_*i*_) < 1) = 1 where *p*(*X*_*i*_) = *P*(*A_i_* = 1|*X_i_*), which means all patients have a strictly positive probability for receiving concordant treatment (*A*_*i*_ = 1).

In addition to the above identifiability assumptions, we made independent censoring (i.e. $C_{i}⫫T_{i}|X_{i}$ *)* and independent entry assumption given the covariates (i.e. $E_{i}⫫T_{i}|X_{i}$).

We adopted two different estimation strategies to obtain the impact of treatment concordance: semi-parametric Cox Proportional Hazard estimator adjusted with a set of measured confounders *X* [32] and non-parametric Kaplan Meier estimator adjusted with Inverse Probability Treatment Weighting (IPTW). We formulated an IPTW, *w*_*i*_ for each sample as $w_{i}=\frac{P\left(A=a_{i}\right)}{P\left(A_{i}=a_{i}|X_{i}\right)}$ [33] and estimated *P*(*A*) non-parametrically and *P*(*A*|*X*) using a logistic regression model (R stats v4.0.2 [56]) in a 10-fold cross-fitting. A set of measured confounders (i.e., *X_i_*) included patients’ sex, age, OncoNPC prediction uncertainty (in entropy of posterior distribution over 22 cancer types), sequencing panel (i.e., OncoPanel) version, mutational burden, CNA burden, subsets of OncoNPC predicted cancer types and metastasis sites, and finally pathological histology (e.g., adenocarcinoma tumor or neuroendocrine tumor). Since patients with CUP who met the treatment criteria (i.e., follow-up start time) but did not receive clinical panel sequencing (i.e., entry time) could not be included in the analysis, we adjusted for the left truncation by defining the risk set ℛ(t) at time *t*, which corresponds to the set of patients followed up in the analysis up to time *t* as follows

$$ℛ(t)=\left\{i|E_{i}\leq t\leq T_{i}\right\}$$,

where *E*_*i*_ is the entry time of patient *i*. With the independent entry assumption as stated before, we obtained survival function from Kaplan-Meier estimator as follows

$$\hat{S}(t)=\underset{i:T_{i}\leq t}{\prod }\left(1-\frac{\sum_{k:T_{k}=T_{i}}w_{k}}{\sum_{j:j\in ℛ\left(T_{i}\right)}w_{j}}\right)$$.

In this formulation, each individual is weighted by the corresponding IPTW, *w*_*i*_, and we obtained two different survival functions for the treatment concordant and discordant groups. The adjusted Kaplan-Meier estimator provides a consistent estimate of impact of the treatment concordance under the assumptions stated above [33]. Once we obtained the survival estimates for the two groups, we used a weighted log-rank test [57] to test for a significant difference in survival.

In the Cox proportional hazard regression framework, we estimated the hazard function of patient *i* as follows: $\lambda \left(t|A_{i},X_{i}\right))=\lambda_{0}(t)\exp\left(\alpha A_{i}+\beta^{T}X_{i}\right)$, where $\alpha ,A_{i}\in \mathbb{R}$ and $\beta ,X_{i}\in {\mathbb{R}}^{m}$ (*m* is the number of measured confounders). Under the above identifiability assumptions and validity of the estimation model, *e*^*α*^ is the hazard ratio capturing the causal effect of the treatment concordance *A*. Finally, under the assumption of no ties between event times across the patients, the parameters *α* and *β* are estimated by maximizing the following partial likelihood

$$L(\alpha ,\beta )=\underset{i:\delta_{i}=1}{\prod }\frac{\exp\left(\alpha A_{i}+\beta X_{i}\right)}{\sum_{j:j\in ℛ\left(T_{i}\right)}\exp\left(\alpha A_{j}+\beta X_{j}\right)}$$

[32].

## Figures and Tables

**Table 1:** Demographic information of the patients and tumor samples across DFCI, MSK, and VICC.

		DFCI	MSK	VICC	DFCI CUP
Number of patients		18,106	15,151	1,310	962
Patients age at sequence (95 % C.I.)		60.7 (60.5 - 60.9)	60.2 (60.0 - 60.4)	58.3 (57.6 - 59.0)	61.9 (61.1 - 62.7)
Sex; male-female ratio		43.8 - 56.2	43.5 - 56.5	44.5 - 55.5	50.0 - 50.0

**Patients ethnicity (proportion %)**					

White		16,105 (88.9 %)	11,575 (76.4 %)	1,089 (83.1 %)	853 (88.7 %)
Black		538 (3.0 %)	866 (5.7 %)	72 (5.5 %)	38 (4.0 %)
Asian		554 (3.1 %)	956 (6.3 %)	17 (1.3 %)	34 (3.5 %)
Hispanic		379 (2.1 %)	744 (4.9 %)	14 (1.1 %)	15 (1.6 %)
Others		530 (2.9 %)	1010 (6.7 %)	118 (9.0 %)	22 (2.2 %)

**Sequenced Tumor Samples**					

Total number of samples		18,816	16,294	1,335	971

**Panel version (proportion %; 95% sequence date range)**					

v1		OncoPanel v1 1,924 (10.2 %; 2013-8-20 - 2014-8-17)	MSK-IMPACT341 1,803 (11.1 %; Not available)	VICC-01-T5A 307 (23.0 %; Not available)	OncoPanel v1 47 (4.8 %; 2013-9-8 - 2014-8-12)
v2		OncoPanel v2 5,304 (28.2 %; 2014-9-28 - 2016-10-5)	MSK-IMPACT410 6,917 (42.5 %; Not available)	VICC-01-T7 1,028 (77.0 %; Not available)	OncoPanel v2 203 (20.9 %; 2014-11-5 - 2016-10-5)
v3		OncoPanel v3 11,588 (61.6 %; 2016-11-11 - 2021-1-6)	MSK-IMPACT468 7,574 (46.5 %; Not available)		OncoPanel v3 721 (74.3 %; 2016-12-14 - 2020-12-23

**Biopsy site type**					

Primary		11,662 (62.0 %)	9,576 (58.8 %)	622 (46.6 %)	.
Metastatic recurrence		5,737 (30.5 %)	6,718 (41.2 %)	637 (47.7 %)	.
Local recurrence		673 (3.6 %)	Not available	64 (4.8 %)	.
Unspecified/others		744 (4.0 %)	Not available	12 (0.9 %)	.
Cancer group	OncoTree Cancer type				*Predicted cancer type*

Lung (Thoracic)	Non-Small Cell Lung Cancer (NSCLC)	3,489 (18.5 %)	3,183 (19.5 %)	137 (10.3 %)	280 (28.8 %)
	Pleural Mesothelioma (PLMESO)	258 (1.4 %)	118 (0.7 %)	2 (0.1 %)	9 (0.9 %)

Gastrointestinal	Colorectal Adenocarcinoma (COADREAD)	2,525 (13.4 %)	1,919 (11.8 %)	232 (17.4 %)	63 (6.5 %)
	Esophagogastric Adenocarcinoma (EGC)	988 (5.3 %)	495 (3.0 %)	59 (4.4 %)	69 (7.1 %)
	Pancreatic Adenocarcinoma (PAAD)	772 (4.1 %)	980 (6.0 %)	53 (4.0 %)	85 (8.8 %)
	Cholangiocarcinoma (CHOL)	241 (1.3 %)	338 (2.1 %)	44 (3.3 %)	33 (3.4 %)
	Gastrointestinal Neuroendocrine Tumors (GINET)	219 (1.2 %)	76 (0.5 %)	18 (1.3%)	46 (4.7 %)
	Pancreatic Neuroendocrine Tumor (PANET)	121 (0.6 %)	133 (0.8 %)	12 (0.9 %)	23 (2.4 %)

Sarcoma	Gastrointestinal Stromal Tumor (GIST)	273 (1.5 %)	217 (1.3 %)	5 (0.4 %)	3 (0.3 %)

Head and Neck	Head and Neck Squamous Cell Carcinoma (HNSCC)	473 (2.5 %)	285 (1.7 %)	20 (1.5 %)	52 (5.4 %)
	Well-Differentiated Thyroid Cancer (WDTC)	166 (0.9 %)	166 (1.0 %)	8 (0.6 %)	1 (0.1 %)

Skin	Melanoma (MEL)	729 (3.9 %)	619 (3.8 %)	187 (14.0 %)	43 (4.4 %)

Breast	Invasive Breast Carcinoma (BRCA)	2,558 (13.6 %)	3,113 (19.1 %)	274 (20.5 %)	85 (8.8 %)

Gynecologic	Ovarian Epithelial Tumor (OVT)	1,213 (6.4 %)	525 (3.2 %)	81 (6.1 %)	58 (6.0 %)
	Endometrial Carcinoma (UCEC)	703 (3.7 %)	703 (4.3 %)	34 (2.5 %)	18 (1.9 %)

Hematologic	Acute Myeloid Leukemia (AML)	150 (0.8 %)	1 (0.0 %)	0 (0.0 %)	1 (0.1 %)
	Non-Hodgkin Lymphoma (NHL)	110 (0.6 %)	88 (0.5 %)	0 (0.0 %)	1 (0.1 %)

Genitourinary	Prostate Adenocarcinoma (PRAD)	601 (3.2 %)	1,222 (7.5 %)	27 (2.0 %)	27 (2.8 %)
	Renal Cell Carcinoma (RCC)	457 (2.4 %)	497 (3.1 %)	39 (2.9 %)	24 (2.5 %)
	Bladder Urothelial Carcinoma (BLCA)	550 (2.9 %)	505 (3.1 %)	41 (3.1 %)	21 (2.2 %)

Neuro	Diffuse Glioma (DIFG)	2,041 (10.8 %)	1,069 (6.6 %)	47 (3.5 %)	25 (2.6 %)
	Meningothelial Tumor (MNGT)	179 (1.0 %)	42 (0.3 %)	15 (1.1 %)	4 (0.4 %)

**Table 2:** Demographic details of patients wit CUP in the concordant and discordant treatment groups.

	Concordant treatment group (n = 77)	Discordant treatment group (n = 81)
Sex; male-female ratio	0.442-0.558	0.556-0.444
Age at seqeuncing (95% C.I.)	64 (61.6 - 66.4)	62 (59.4 - 64.6)
Prediction uncertainty (in entropy; 95% C.I.)	0.550 (0.426 - 0.675)	0.988 (0.850 - 1.127)

**OncoPanel version (proportion in %)**		

v1	1 (1.30%)	1 (1.24%)
v2	9 (11.7%)	15 (18.5%)
v3	67 (87.0%)	65 (80.2%)

Mutational burden (95% C.I.)	0.027 (0.021 - 0.033)	0.033 (0.027 - 0.040)
CNA burden (95% C.I.)	0.201 (0.166 - 0.236)	0.186 (0.155 - 0.217)

**Predicted primary cancer groups (proportion in %)**		

Lung	15 (19.5%)	24 (29.6%)
Breast	5 (6.50%)	11 (13.6%)
GI	33 (42.9%)	16 (19.8%)
Gyn	9 (11.7%)	5 (6.17%)
Others	15 (19.5%)	25 (31.0%)

**Metastatic sites (proportion in %)**		

Brain	4 (5.20%)	8 (9.88%)
Bone	7 (9.10%)	10 (12.3%)
Soft tissue	6 (7.79%)	5 (6.17%)
Others	60 (77.9%)	58 (71.6%)

**Histology (proportion in %)**		

Adenocarcinoma	41 (53.2%)	32 (39.5%)
Neuroendocrine	9 (11.7%)	11 (13.6%)
Squamous cell	2 (2.60%)	6 (7.41%)
Others	25 (32.5%)	32 (39.5%)

Treatment start date (95% C.I.)	2018-4-30 (2017-12-24 - 2018-9-3)	2018-3-1 (2017-10-28 - 2018-7-3)

## Data Availability

The multicenter NGS tumor panel sequencing data is available upon request at the AACR Project GENIE website: https://www.aacr.org/professionals/research/aacr-project-genie/. The processed somatic variants data from Profile DFCI and de-identified clinical data utilized in the treatment concordance analysis are available in https://github.com/itmoon7/onconpc.
